# Functional electrical stimulation through direct 4-channel nerve stimulation to improve gait in multiple sclerosis: a feasibility study

**DOI:** 10.1186/s12984-015-0096-3

**Published:** 2015-11-14

**Authors:** Janet Hausmann, Catherine M. Sweeney-Reed, Uwe Sobieray, Mike Matzke, Hans-Jochen Heinze, Jürgen Voges, Lars Buentjen

**Affiliations:** Department of Neurology, Otto-von-Guericke University, Magdeburg, Leipziger Str. 44, 39120 Magdeburg, Germany; Department of Stereotactic Neurosurgery, Otto-von-Guericke University, Magdeburg, Leipziger Str. 44, 39120 Magdeburg, Germany; German Center for Neurodegenerative Diseases (DZNE), Site Magdeburg, Leipziger Str. 44, Magdeburg, Germany

**Keywords:** Functional electrical stimulation, Multiple sclerosis, Peroneal nerve, Central drop foot, Hemiparesis, Rehabilitation, Gait, Gait improvement, Neuroprosthetics, Quality of life

## Abstract

**Background:**

Gait dysfunction due to lower limb central paralysis, frequently involving drop foot, is a common cause of disability in multiple sclerosis and has been treated with transcutaneous functional electrical stimulation (FES). We provide here the first report of 4-channel semi-implantable FES of the peroneal nerve which has been successfully used for rehabilitation in patients following stroke.

**Methods:**

FES was implemented via a 4-channel semi-implantable closed-loop system (ActiGait®, ©Ottobock), generating dorsiflexion in drop foot. Walking distance, gait symmetry (temporospatial gait analyses, Vicon Motion Systems®), gait velocity (10 m walking test) and quality of life (SF-36 questionnaire) were measured to evaluate the therapeutic benefit of this system in two patients with progressive MS.

**Results:**

Walking distance increased from 517 to 1884 m in Patient 1 and from 52 to 506 m in Patient 2. Gait velocity did not change significantly in Patient 1 and increased from 0.6 to 0.8 m/s in Patient 2. Maximum deviations of center of mass from the midline to each side changed significantly after 3 months of stimulation compared to baseline, decreasing from 15 to 12 mm in Patient 1 and from 47 to 37 mm in Patient 2. Both patients experienced reduced pain and fatigue and benefits to quality of life. Adverse events did not occur during the observation period.

**Conclusion:**

We conclude that implantable 4-channel FES systems are not only feasible but present a promising new alternative for treating central drop foot in MS patients.

## Background

Multiple sclerosis (MS) is an autoimmune-mediated disorder leading to progressive neurodegeneration, with increasing disability in many patients despite modern treatment approaches. Although recent surveys have demonstrated the importance of walking for MS patients [1], gait dysfunction occurs in 80 % of all MS patients after a 10 to 15 year disease duration [2], often due to central paralysis of the lower limbs, and commonly involving a drop foot. Gait disturbances can lead to frequent falls [3] and a pathological compensatory gait [4]. Common treatment options such as walking aids, ankle foot orthoses (AFO) to prevent forefoot floor contact during the swing phase of walking, and pharmacological treatments (such as the potassium channel blocker fampridine and local or systemic antispastic therapy) show only limited efficacy, with benefits only in individual cases.

Functional electrical stimulation (FES) was first proposed in the 1960s as a stroke therapy [5]. It describes application of an electric current to a nerve in order to induce muscle contraction and thereby assist in performance of a functional activity such as walking. The ActiGait® System (details of the operation mode are described below) was officially approved in Germany in 2007 for hemiplegia following ischaemic or hemorrhagic stroke. Because the ActiGait System has been recently introduced, many studies investigating its use have not yet reached publication. We therefore enquired directly with Ottobock, who reported that the device has been implanted in approximately 400 post-stroke patients (effective July 2015), but that implantation for other indications remains rare.

Therapeutic success may be anticipated, however, in upper motor neuron damage regardless of etiology [6]. Moreover, benefits have been reported in MS patients either receiving surface FES [7–11] or by use of a 2-channel implanted system that stimulates the two branches of the common peroneal nerve [12]. Difficulties reported by some of our patients in handling a surface FES system, and sensory side-effects resulting from external attachment of the electrodes, led us to investigate whether the advantages offered by an implantable system could include reduced sensory side-effects. We provide here the first report of successful implementation of FES applied directly to the peroneal nerve via an implanted 4-channel cuff electrode to aid dorsiflexion in MS in two patients. Both patients, suffering from progressive disease and central drop foot, experienced considerable improvement in gait physiology, with significantly increased walking distance, as well as significant enhancement of quality of life (QoL).

## Methods

### Participants

An electrode cuff for FES of the peroneal nerve (ActiGait®, ©Ottobock) was implanted in two patients suffering from foot drop secondary to MS. Approval by the local ethical review committee and informed consent regarding off-label use of the device was obtained beforehand.

### Patient 1

Patient 1 was a 53-year-old lady who was diagnosed with MS in 2002 after developing right-sided hemihypesthesia, and followed a relapsing-remitting course. In retrospect, a transient sensory disturbance caudal from dermatome T10 in 1992 was in fact the first episode. A further episode, involving numbness of the right foot, followed in 2002. A slowly advancing, persistent mild paresis of the left leg affecting dorsiflexion and eversion of the foot began in December 2005. Ankle-twisting with increasing walking distance limited her walking distance to 500 m. The patient also suffered from temporary episodes of general fatigue. The disease evolved into a progressive course without further symptom remission. The Expanded Disability Status Scale (EDSS) [13] score was 3.5/10. The diagnosis of MS was supported by cerebral MRI lesions and delayed somatosensory evoked potentials (SSEP).

Immunomodulatory treatment with Interferon beta-1a (Avonex, Biogen Idec) was administered from 2003–2013, then discontinued following 8 years without exacerbations and increasing needle phobia. Symptomatic treatment of gait disturbance with Fampridine retard 2x 10 mg (Fampyra, Biogen Idec) from 2011 led to a more fluent gait pattern and facilitated stair climbing. Despite weekly physiotherapy, the drop foot remained impairing and walking distance did not improve. Because the movement limitations were only debilitating over longer distances, an AFO or other walking aid were not deemed to offer benefits outweighing their inconvenience.

At referral in April 2013, walking required considerable concentration, with frequent stumbling due to forefoot catching and ankle twisting, especially with increasing walking distance. After walking 500 m, the patient suffered from left leg pain. Neurological examination of motor skills revealed a mild distal left hemiparesis, particularly of the lower limb, with mild spasticity. The passive dorsiflexion/plantar flexion range exceeded 30° with a leg-foot-angle of approximately 90° in maximum passive dorsiflexion in a stretched leg position. Muscle strength in the left leg, rated according to the British Medical Research Council (BMRC) criteria, was as follows: hip flexion 4/5, knee flexion 4/5, other proximal movements 5/5, ankle dorsiflexion 1/5, pronation 2/5, supination 4/5, plantar flexion 5/5. Sensory system examination revealed hypesthesia of the left lower leg and foot, with pallhypesthesia of 5/8 over the left and 8/8 over the right malleolus, measured using a Rydel Seiffer tuning fork, Position-, temperature- and pain sensation were intact. Her gait pattern was spastic-ataxic but narrow-based. The outer edge of the foot dropped during the swing phase, with the forefoot dragging over the floor with increasing walking distance. Mild spasticity of the toes was present, with minimal ankle joint instability and the gait was asymmetrical.

A four-week test phase with surface FES increased walking distance and reduced effort on walking, but sensory side-effects were not well-tolerated. We therefore implanted an electrode cuff for FES of the left peroneal nerve (ActiGait®, ©Ottobock) in September 2013. There were no peri- or post-operative complications. After a healing phase of 3 weeks, we activated the stimulation system.

### Patient 2

Patient 2 was a 46-year-old man, diagnosed with MS with a primary progressive course in 2007. He developed a slowly deteriorating paresis of the right leg with a disabling paresis of dorsiflexion (EDSS 6.5). With hindsight, the first symptoms appeared in 1998, with right upper limb weakness and numbness. Also of note is a spinal disc herniation of lumbar disc 4/5 to the right side, surgically treated in 2008.

On initial presentation in 03/2013, he reported a walking distance-dependent physical fatigue with increasing right leg weakness and forefoot catching, with concomitant back, sacral, and pelvic pain. Concentration was necessary to avoid stumbling, and he required a cane and an AFO as walking aids. Fatigue limited walking distance to 50 m using the AFO, and he was unable to walk without the AFO due to ankle twisting with each step.

Clinical examination revealed mild right hemiparesis and moderate lower limb spasticity with contracture of the calcaneal tendon. Passive dorsiflexion/plantar flexion range exceeded 30°, with a leg-foot-angle of approximately 90° on maximum passive dorsiflexion in a stretched leg position. Muscle strength in the right leg, according to BMRC criteria, was as follows: hip flexion 3/5, extension 4+/5, abduction and adduction 5/5, knee flexion 2/5, extension 5/5, ankle dorsiflexion 1/5, pronation and supination 2/5, plantar flexion 4+/5. Sensory examination revealed no sensory deficits in the lower limbs. His gait pattern was spastic-ataxic with circumduction of the right leg, initial forefoot-floor-contact, and dropping of the forefoot and outer edge during the swing phase. After a few steps, he began to drag his right leg, followed by the onset of spasticity of the toes and instability in the ankle and knee joints. As for Patient 1, MRI and electrophysiological findings supported the diagnosis of MS.

Immunomodulatory therapy was conducted with Interferon beta-1b 0.25 mg/ml (Betaferon, Bayer Health Care) every other day from diagnosis. He received no antispastic treatment. A temporary treatment with Fampridine retard 2x 10 mg (Fampyra, Biogen Idec) did not improve walking with the cane and AFO.

Although we observed a clinical benefit following a 4-week test phase with surface FES, the patient experienced difficulties with exact electrode positioning, and moreover, the electrodes were easily dislodged during walking. Surface FES was therefore deemed unsuitable for daily use, resulting in the decision to employ an implanted FES system for direct stimulation of the right peroneal nerve. We implanted the ActiGait® system in November 2013 without complications and activated it 5 weeks later.

### FES system

**Fig. 1:**
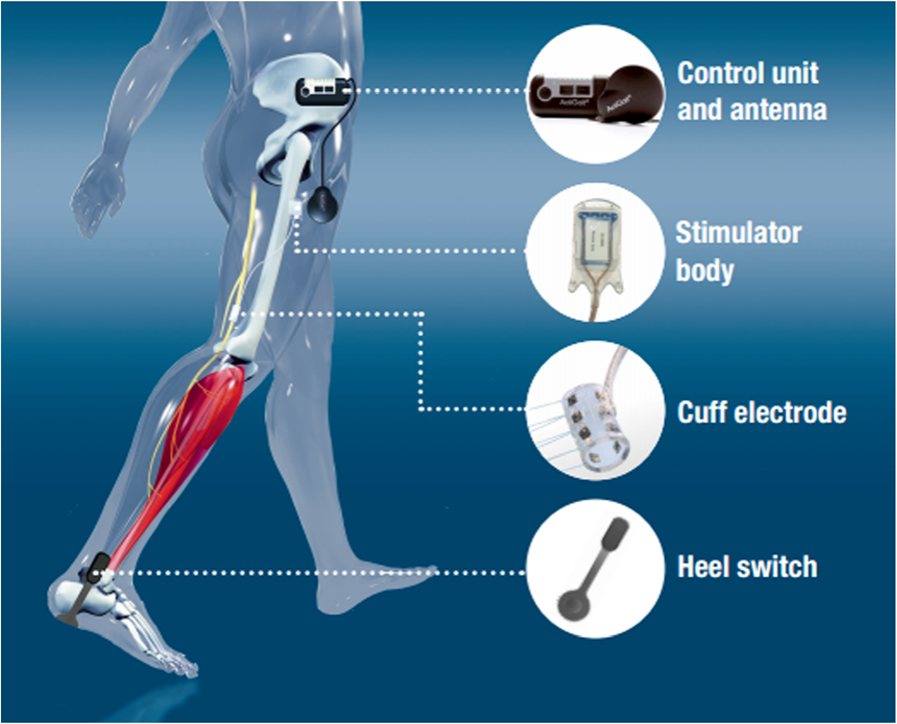
Components of the ActiGait stimulation system. The control unit worn around the patient’s waist receives a trigger signal wirelessly from the externally placed heel switch when heel lift is registered. It generates a variable electromagnetic field in the coiled antenna, which is connected to it. Transcutaneous electromagnetic induction is used to transfer the power and control signals to the implanted stimulator, which generates the stimulation pulses in 4 independent current sources. These impulses are then delivered through a dual lumen cable to 4 circularly arranged sets of electrodes within a 23 mm silicon cuff electrode, and selectively stimulate the fascicles of the common peroneal nerve and thus trigger balanced dorsiflexion of the foot

ActiGait® (©Ottobock) is a semi-implantable closed-loop FES system generating dorsiflexion in drop foot (see Fig. 1). The system is adapted to individual gait phase and velocity by registering the patient’s heel lift through an externally placed heel switch. The control unit worn around the patient’s waist receives this trigger signal wirelessly and generates a variable electromagnetic field in the coiled antenna, which is connected to it. Transcutaneous electromagnetic induction is used to transfer the power and control signals to the implanted stimulator, which generates the stimulation pulses in 4 independent current sources. These impulses are then delivered through a dual lumen cable to 4 circularly arranged sets of electrodes embedded within a 23 mm silicone cuff. Each of the 4 channels can be controlled independently of the other channels, thus enabling the programmer to control the volume of tissue activated within the nerve. The fascicles of the common peroneal nerve can thus be selectively stimulated to trigger a balanced dorsiflexion of the foot while avoiding stimulation of sensory fascicles.

### Assessment tools

Evaluation of gait symmetry, comprising walking distance measurement and gait velocity, as well as quality of life (QoL), using the SF-36 questionnaire [14], were performed before implantation of the electrode, after the stimulation was first commenced (referred to as “activation day”), and again 3 months later.

We used the 3D-Kinematicsystem from “Vicon Motion Systems, Oxford UK” to capture the kinematics, required for gait velocity measurement and for calculation of the patient’s center of mass to quantify gait symmetry. Markers were placed on the head, trunk, and limbs in accordance with the Plug-In Gait software package, which corresponds with the clinical gold standard in gait analysis. To avoid inclusion of acceleration and braking phases in the gait velocity measurements, the walking space provided for patients had a total length of 8 meters, suitable for recording a walking distance of at least 4 meters. In order to maintain balance with an asymmetric gait, trunk, limb and head movements may be employed, stabilizing the gait by shifting the center of mass [15]. The change in gait resulting from stimulation can be quantified by calculating the shift in the patient’s center of mass after stimulation. The motion capture system allows the patient’s center of mass to be calculated, based on the position locations of the markers in 3-D space, thus providing a holistic view of the body as a single moving system in equilibrium in the transverse plane [16] and thereby a measurement of the effect of the stimulation on gait. Total walking distance in meters was measured in the clinic corridor. The patients were asked to walk until they no longer felt able to continue. Gait velocity was defined as speed over a distance of 10 meters, measured three times with and without stimulation. Both examinations took place on different days to avoid bias of results by fatigue. Gait velocity and gait analysis were performed successively with a pause of at least 30 min for recovery.

### Statistics

T-tests were applied to provide a quantitative indicator of the significance of the changes in the assessment measures following stimulation treatment. The two measurements of walking distance on activation day, first without, then during stimulation, were compared with the two measurements performed 3 months later. The three measurements of gait velocity performed on activation day before stimulation was commenced were compared with three measurements made 3 months later during stimulation. The difference between these values reflects both the efficacy of the device and the improvement made over the time of its use. Simulation supports the validity of T-tests with low sample numbers, defined as *N* = 2 to 5. [17]. The QoL questionnaire, completed before stimulation commencement and repeated following 3 months of stimulation treatment, consists of 36 items, each rated on a scale from 0 to 100. In order to provide an indication of whether QoL was generally improved following treatment, each of the 36 items was taken as a separate indicator, providing 35° of freedom.

We measured the maximum deviations of center of mass to each side from the midline for each gait cycle pre-operatively and 3 months post-operatively. For Patient 1, two gait cycles were completed at each assessment (*N* = 4, including both sides), and Patient 2 completed 3 gait cycles (*N* = 6, including each side). The absolute difference between the two sets of distance measures was subjected to a *T*-test of the null hypothesis that the deviations from the midline post-operatively did not differ from those measured pre-operatively.

## Results

### Patient 1

**Fig. 2:**
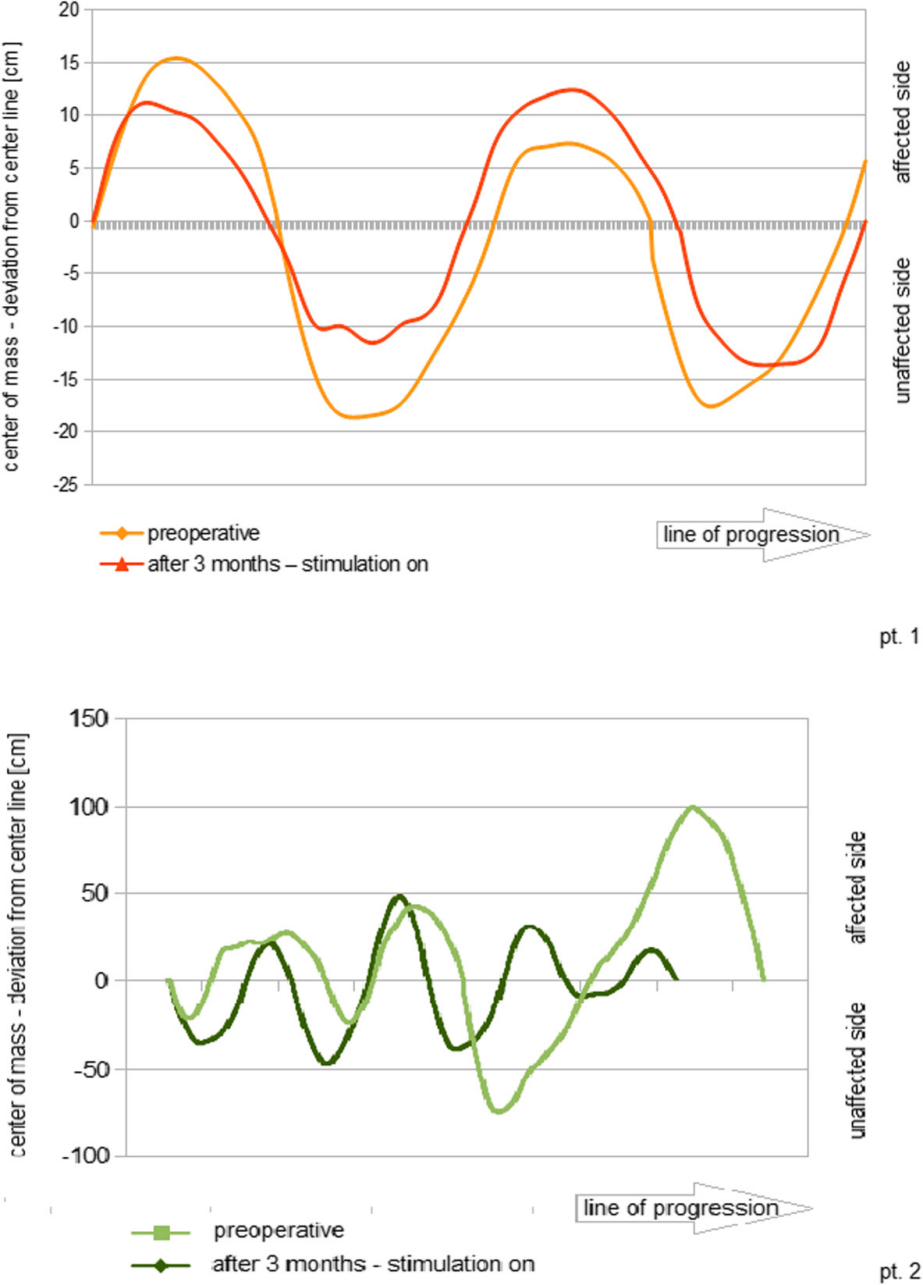
Undulation of center of mass along the line of progression. To evaluate gait symmetry, we examined the undulation of center of mass along the progression line with a temporospatial gait analysis. Preoperatively, the center of mass was located on the non-paretic side of the body, and the position of the center of mass moved to the left and right sides with a great amplitude, with varying degrees of severity, in both patients. In both patients gait symmetry increased after 3 months under stimulation, with less drift to both sides and a shift of the center of mass to the center of the body while walking

In Patient 1, with stimulation switched on, balanced dorsiflexion was obtained in the ankle joint without sensory side-effects. Dorsiflexion was adequate to prevent contact between the outer edge of the foot and the floor during the swing phase. A plain stabilization of ankle and knee joint during the stance phase was already apparent on first activation of the system. Hyperflexion tending to genu recurvatum no longer occurred. After 3 months, the patient reported an improvement in walking and an increased sense of security. With increasing walking distance (more than 500 m), the benefits of stimulation on walking became more evident. She demonstrated a nearly symmetrical gait pattern under stimulation. The drift to each side on gait analysis measured post-operatively during stimulation differed significantly from pre-operative measurements (one-sided paired *T*-test: T = 5.24, *p* = 0.014) with an average decrease from 15 to 12 mm (Fig. 2). Moreover, it may be observed that the centre of mass was more central post-operatively. Without stimulation, her gait pattern remained nearly unchanged compared with preoperatively, though the ataxic component was less distinct.

**Fig. 3:**
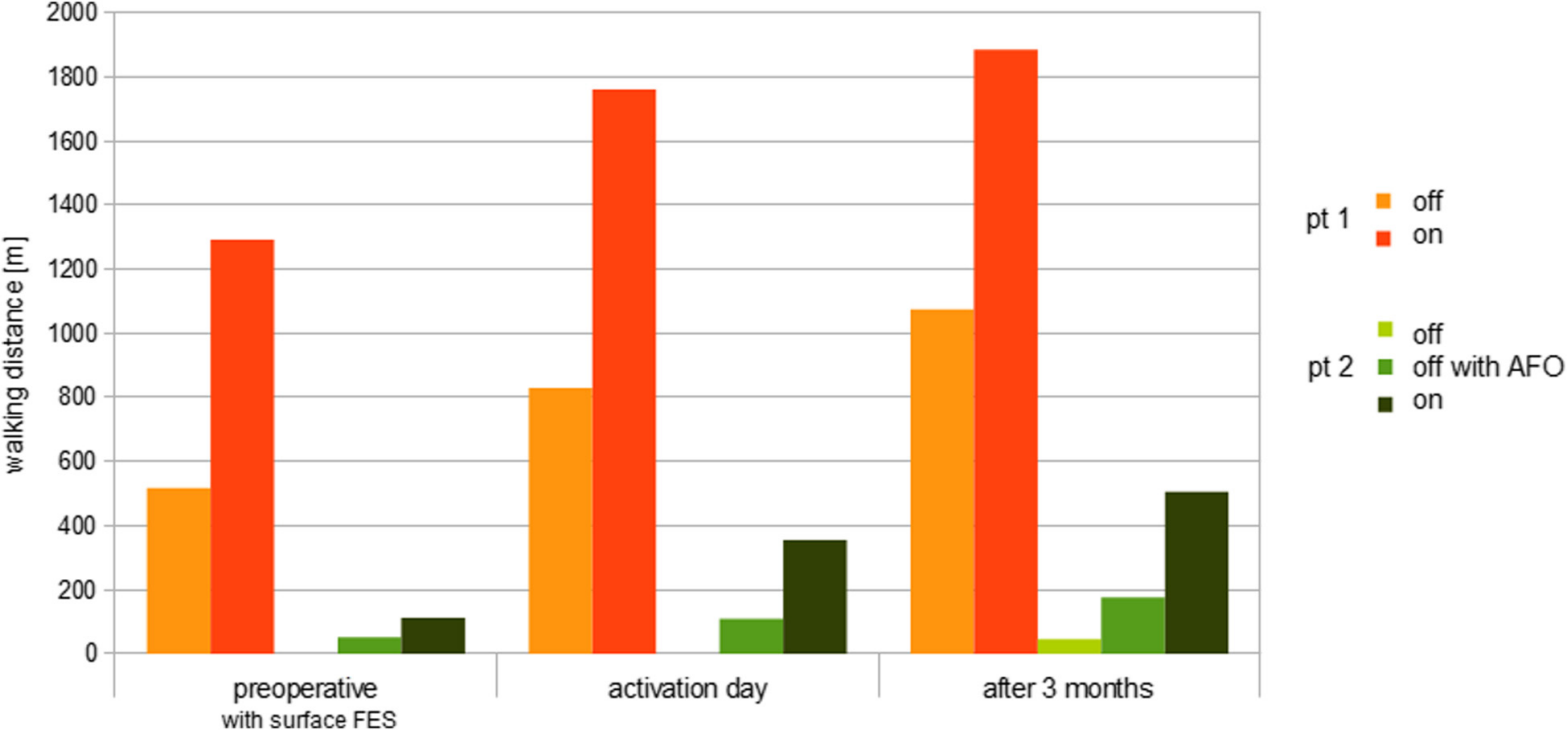
Walking distance in longitudinal follow up. Walking distance under different conditions preoperatively, at activation day and after 3 months. Stimulation therapy led to increased walking distance in both patients. In patient 1 walking distance increased from 517 m to 1884 m after 3 months of stimulation (orthotic effect of FES). Moreover, the patient could walk 1075 m when stimulation was turned off, suggesting a therapeutic effect of FES in addition. Postoperatively, walking distance was significantly increased in patient 1 with the stimulation activated compared with off (one-sided paired *T*-test: T = 14.3, *p* = 0.022). In patient 2 maximum walking distance improved from 52 m preoperative with an AFO to 506 m after 3 months of using FES and to 176 m with AFO. Postoperatively, walking distance was significantly increased with the stimulation activated compared with off (one-sided paired *T*-test: T = 7.85, *p* = 0.040) and with AFO in the absence of stimulation (one-sided paired *T*-test: T = 6.86, *p* = 0.046). Remarkably, the patient was able to walk a distance of 46 m without an AFO or FES 3 months after activation

Walking distance increased more than threefold from 517 m to 1884 m after 3 months of stimulation (orthotic effect of FES). Moreover, the patient could walk 1075 m when stimulation was turned off, suggesting a therapeutic effect of FES in addition (Fig. 3). Postoperatively, walking distance was significantly increased with the stimulation activated compared with off (one-sided paired *T*-test: T = 14.3, *p* = 0.022). There was no significant change in maximum gait velocity over 10 m (one-sided paired *T*-test: T = 1.6, *p* = 0.13; Table 1).

**Table 1:** Gait velocity under different stimulation conditions. Table 1 shows gait velocities in patient 1 and patient 2 on activation day and after 3 months. In patient 1 there was no significant change in maximum gait velocity over 10 m (one-sided paired *T*-test: T = 1.6, *p* = 0.13). In patient 2, however, maximum gait velocity had increased significantly immediately after activation, persisting after 3 months of FES, and on and off mode also differed significantly at each time point (one-sided paired *T*-test: T = − 8.9, *p* = 0.006)

Velocity [m/s]	Patient 1		Patient 2		
	off	on	off	off using AFO	on
Activation day	1.7	1.7	–	0.6	0.8
After 3 months	1.4	1.5	0.6	0.8	0.8

**Fig. 4:**
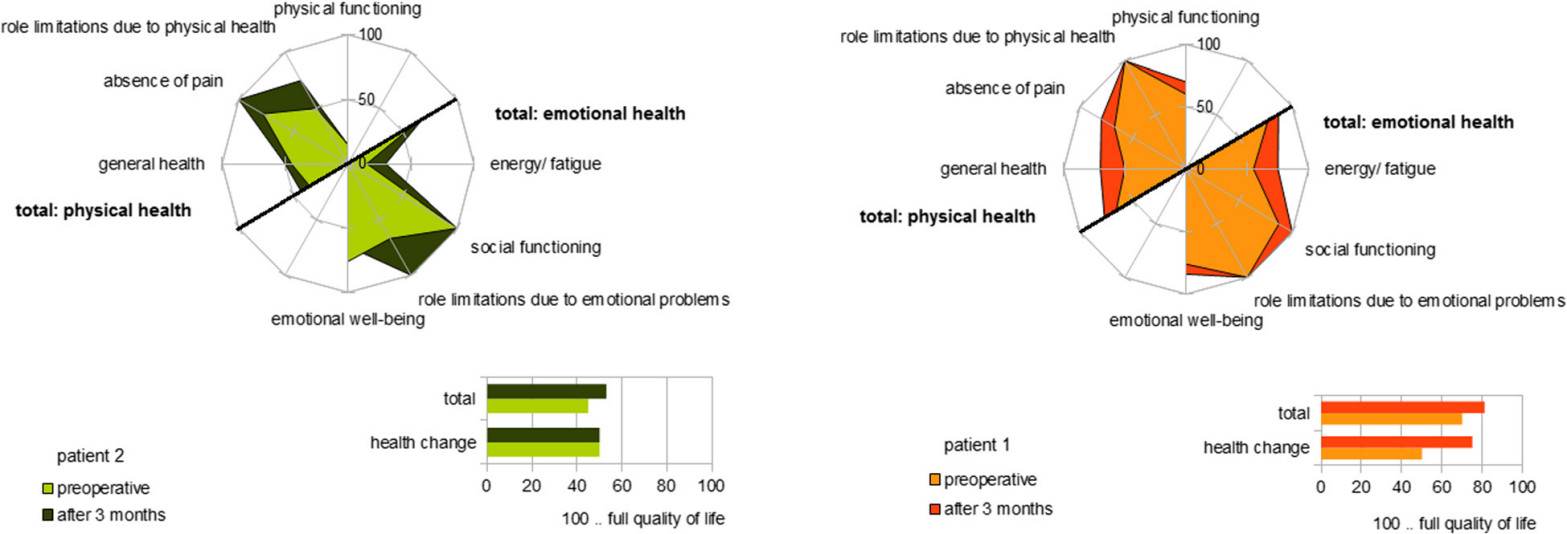
Quality of life using SF-36 questionnaire. Different parameters of quality of life are presented with a radar chart. We see different patterns of changes in both patients, but consistent improvements in both in physical, emotional and general health, absence of pain and benefits in energy and fatigue. Bar charts show total scores of the SF-36 questionnaire with significant improvements compared to preoperative statements in both patients (one-sided paired *T*-test; pt. 1: T = −3.2, *p* = 0.0015; pt. 2: T = −1.7, *p* = 0.048)

Evaluation of QoL parameters using an SF-36 questionnaire revealed various improvements compared with preoperative statements (one-sided paired *T*-test: T = −3.2, *p* = 0.0015), especially pertaining to parameters of physical as well as emotional health; in particular pain and fatigue were reduced. The patient reported a marked change in general health (Fig. 4). Patient 1 has received an invalidity pension since having bronchial carcinoma in 1992, so effect on occupation was not assessed.

### Patient 2

In patient 2, ankle stability improved markedly, and walking was facilitated, including an ability to cover longer distances. After 3 months, walking-dependent pain had subsided. The patient was able to resume his profession in food control, working in field service 3 days a week. Maximum walking distance improved from 52 m preoperatively with an AFO to 506 m after 3 months of using FES (Fig. 3). Postoperatively, walking distance was significantly increased with the stimulation activated compared with off (one-sided paired *T*-test: T = 7.85, *p* = 0.040) and with an AFO in the absence of stimulation (one-sided paired *T*-test: T = 6.86, *p* = 0.046). A walking distance of 176 m remained after turning the system off. The EDSS score was improved by 0.5 points. Remarkably, the patient was able to walk a distance of 46 m without an AFO or FES 3 months after activation (Fig. 3). Maximum gait velocity had increased from 0.6 to 0.8 m/s immediately after activation, persisting after 3 months of FES, and velocity during the on versus off mode differed significantly (one-sided paired *T*-test: T = − 8.9, *p* = 0.006; Table 1). The drift of center of mass from the midline 3 months after commencement of treatment showed a trend to a reduction in patient 2 (47 vs. 37 mm; one-sided paired *T*-test: T = 2.44, *p* = 0.059).

SF-36 questionnaire evaluation demonstrated improvements in physical and emotional health (one-sided paired *T*-test: T = −1.7, *p* = 0.048). The patient reported reduced pain and fatigue, and experienced fewer physical, health-related role limitations on resuming work (Fig. 4).

## Discussion

Dropping of the forefoot in the swing phase of gait can cause stumbling and falling [18]. A central drop foot is often accompanied by ankle instability simply during standing, as well as in the stance phase of the gait, increasing the risk of the ankle twisting, as commonly observed clinically. Moreover, walking becomes effortful and slow, potentially shortening walking distance [19, 20]. Degenerative joint or back pain due to compensatory weight shift may develop in the long term, as observed in Patient 1.

Patients with drop foot often depend on walking aids such as an AFO, which acts passively as a cast. The main disadvantage in using an AFO is that it does not cause muscle contraction and can thus lead to further joint immobilization. It also lacks the sensory feedback of muscle contraction compared with FES. Moreover, spasticity of the toe flexors, as experienced by Patient 2, can cause pressure sores. FES via surface or implantable electrodes offers a means of compensating for these issues. The few studies reported evaluating implantable FES systems found improvements in gait parameters and QoL in post-stroke patients [21–23]. The ActiGait® system has been employed in a number of studies to date, and few adverse events have occurred [21, 24]. Although the motor response is similar using surface compared with implanted FES [12], the potential risks of surgery may be outweighed by several advantages offered by implanted over surface FES. Firstly, the electrode cuff, with its 4 channels placed circularly around the target nerve, generates the required motor response more precisely than surface stimulation. Because 4 potential channels are available for treatment, an optimum balance between dorsiflexion and eversion for the individual patient can be achieved. In some cases, for instance, a better orthotic effect, with greater ankle joint stability, can be obtained by distinct eversion. Secondly, the patient is not required to apply the electrode to the leg, which takes time, and moreover can be difficult to perform correctly, as reported by patient 2. The use of an implantable system could thus potentially lead to more consistent use of the device, with a concomitant increase in the therapeutic effect. Thirdly, discomfort due to electrical stimulation is minimal, because applying the current directly to the nerve avoids stimulation of non-target nerves, eliminating sensory side-effects, which cannot be achieved using a skin- mounted electrode. FES with an implantable multichannel system can therefore be more comfortable, as experienced by patient 1.

We provide here the first report of an implantable 4-channel FES system in patients with MS. A 2-channel system was implanted in 46 patients with central drop foot of mixed etiology, including 17 MS patients, and provided promising results, which have thus far been presented as preliminary findings [12]; gait performance improvement was similar to that provided in the same patients using a surface stimulation system. The method of fixation, however, whereby the electrode is sewn directly onto the nerve, resulted in considerable adverse effects, with temporary nerve dysfunction in 10 cases, implant failure in 6 cases, and one infection resulting in 4 explantations [12]. The authors report improved results with reduced sutures [12], and preliminary long-term findings were reported in a recent conference, whose proceedings are not yet available [25]. The benefits in terms of gait parameters, QoL, and fatigue reduction found in the few studies investigating surface FES in MS patients were similar to those reported in stroke studies [7–11]. Moreover, the approach compared favorably with the current gold standard of AFO, both in terms of subjective patient reporting and measureable outcomes of improved walking distance and gait symmetry. These findings are consistent with randomized controlled trials comparing AFO with implanted FES post-stroke that demonstrated comparable improvement in gait and QoL [26] as well as superiority of FES for obstacle avoidance [27].

Our patients demonstrate that implantable FES can lead to considerable improvements in gait parameters and QoL. Over the short 3-month observation period, the patients showed an orthotic effect as well as a rehabilitation benefit on walking distance, and increased walking distance impacted QoL considerably. The second patient was able to walk over 500 m with the stimulation system switched on, compared with 50 m before implantation, enabling the patient to walk outside the home and to resume employment. Both patients reported decreased pain, which may result from reduced abnormal joint loading due to pathological compensatory gait patterns and effort in ambulation. In patient 1, although the more balanced and centered gait pattern was observable clinically, the improvement in gait symmetry measurement was less marked than in patient 2. With temporospatial gait analysis, we sought to quantify our clinical observations using a readily measurable component of gait symmetry and found a small but nonetheless statistically significant reduction in drift of center of mass to each side. In patient 1, the increase in walking distance was the more pronounced improvement. In patient 2, the more marked reduction in drift is likely to be because this patient showed a more pronounced asymmetry of gait before the intervention. The difference in findings between these two patients, both of whom reported gait improvement following stimulation treatment, underlines the importance of using a range of outcome measures in quantitative outcome assessment.

Both patients experienced reduced fatigue despite being more active. Social factors also improved, possibly due to increased self-sufficiency and participation, resulting from increased mobility. The current standard therapy for drop foot is an AFO. Our second patient reported an improved walking experience over longer distances with FES in comparison with the AFO, resulting in increased walking distance and gait symmetry. Recent randomized controlled trials comparing AFO with FES, including both surface as well as implanted FES in post-stroke patients, showed FES to provide equivalent improvement in gait and QoL to AFO [26] and enhanced obstacle avoidance ability [27]. In another study, stroke patients evaluated surface FES in a structured interview as generally superior to different kinds of AFO [28], but MS patients only reported advantages from FES in terms of increased walking distance, fitness and physical activity [29]. These findings are consistent with the statements from our second patient, who used an AFO before receiving FES.

After 3 months using the FES, the second patient was able to walk a distance without technical assistance that he could only achieve with the use of an AFO pre-operatively. This improvement is consistent with findings using surface FES, which show that stimulation may lead to enhancement of motor cortex neuroplasticity [30]. It is moreover likely that the regular muscle contraction brought about during the use of the FES led to improvements in muscle strength, which do not occur when the ankle is held in a fixed position with an AFO.

The progressive character of MS may be considered as a critical difference between post-stroke and MS patients. However, patients who have suffered a stroke are recognized to be at risk of further cerebrovascular events due to the persistence of the underlying pathology, such as arteriosclerosis or atrial fibrillation. Even if a further episode leads to persisting or additional deficits, the patient may still benefit from assisted dorsiflexion of the foot. If motor function of the ipsilateral leg diminishes, ActiGait® allows adaptation of stimulation parameters, for example to increase the amplitude of dorsiflexion. Many MS patients also remain stable for long time periods due to new and effective immunomodulatory basis treatment [31], and may therefore also be suitable candidates to receive an implantable FES system. The disadvantages of surface over implantable FES, such as poor tolerance of sensory effects, and difficulties with electrode placement, thus support the use of implantable rather than surface FES, even in progressive disorders.

## Conclusion

We conclude that implantable FES systems are a feasible new option for treating central foot drop in MS patients. We demonstrate improvements in gait parameters, and reduction of pain and fatigue, as well as positive changes in quality of life in two individuals suffering from progressive MS treated with an implantable device for FES of the peroneal nerve. The 4 channel cuff FES system appears to offer the recognized advantages of an implantable system compared with surface stimulation and moreover offers the possibility of achieving these benefits with fewer side-effects and complications than those encountered using a system in which the electrodes are sewn directly onto the nerve. The success of the therapy in this preliminary study paves the way for a larger trial to evaluate the benefits and general safety of the approach.

## Abbreviations

AFO: Ankle foot orthosis
EDSS: Expanded disability status scale
FES: Functional electrical stimulation
MRI: Magnetic resonance imaging
MS: Multiple sclerosis
Pt.: Patient
QoL: Quality of life
SSEP: Somatosensory evoked potentials
